# A Longitudinal Study of Mitral Regurgitation Detected after Acute Myocardial Infarction

**DOI:** 10.3390/jcm11040965

**Published:** 2022-02-13

**Authors:** Harish Sharma, Mengshi Yuan, Iqra Shakeel, James Hodson, Ashwin Radhakrishnan, Samuel Brown, John May, Kieran O’Connor, Nawal Zia, Sagar N. Doshi, Sandeep S. Hothi, Jonathan N. Townend, Saul G. Myerson, Peter F. Ludman, Richard P. Steeds, M. Adnan Nadir

**Affiliations:** 1 Institute of Cardiovascular Sciences, University of Birmingham, Birmingham B15 2TT, UK; a.radhakrishnan@nhs.net (A.R.); sagar.doshi@uhb.nhs.uk (S.N.D.); s.hothi@nhs.net (S.S.H.); john.townend@uhb.nhs.uk (J.N.T.); peter.ludman@uhb.nhs.uk (P.F.L.); rick.steeds@uhb.nhs.uk (R.P.S.); adnan.nadir@uhb.nhs.uk (M.A.N.); 2 Department of Cardiology, University Hospitals Birmingham, Birmingham B15 2TH, UK; mengshi.yuan@nhs.net (M.Y.); johnmay6@hotmail.com (J.M.); kieran.t.oconnor@gmail.com (K.O.); 3 Medical and Dental School, University of Birmingham, Birmingham B15 2TT, UK; ixs726@student.bham.ac.uk (I.S.); sjb767@student.bham.ac.uk (S.B.); nxz654@student.bham.ac.uk (N.Z.); 4 Institute of Translational Medicine, University Hospitals Birmingham, Birmingham B15 2TH, UK; james.hodson@uhb.nhs.uk; 5 Department of Health Informatics, University Hospitals Birmingham, Birmingham B15 2TH, UK; 6 Department of Cardiology, Royal Wolverhampton NHS Hospitals Trust, Wolverhampton WV10 0QP, UK; 7 Division of Cardiovascular Medicine, Radcliffe Department of Medicine, University of Oxford, Oxford OX3 9DU, UK; saul.myerson@cardiov.ox.ac.uk

**Keywords:** mitral regurgitation, myocardial infarction, serial echocardiography

## Abstract

Background: Mitral regurgitation (MR) is common following myocardial infarction (MI). However, the subsequent trajectory of MR, and its impact on long-term outcomes are not well understood. This study aimed to examine the change in MR severity and associated clinical outcomes following MI. Methods: Records of patients admitted to a single centre between 2016 and 2017 with acute MI treated by percutaneous coronary intervention (PCI) were retrospectively examined. Results: 294/1000 consecutive patients had MR on baseline (pre-discharge) transthoracic echocardiography (TTE), of whom 126 (mean age: 70.9 ± 11.4 years) had at least one follow-up TTE. At baseline, most patients had mild MR (*n* = 94; 75%), with *n* = 30 (24%) moderate and *n* = 2 (2%) severe MR. Significant improvement in MR was observed at the first follow-up TTE (median 9 months from baseline; interquartile range: 3–23), with 36% having reduced severity, compared to 10% having increased MR severity (*p* < 0.001). Predictors of worsening MR included older age (mean: 75.2 vs. 66.7 years; *p* = 0.003) and lower creatinine clearance (mean: 60 vs. 81 mL/min, *p* = 0.015). Change in MR severity was significantly associated with prognosis: 16% with improving MR reached the composite endpoint of death or heart failure hospitalisation at 5 years, versus 44% (*p* = 0.004) with no change, and 59% (*p* < 0.001) with worsening MR. Conclusions: Of patients with follow-up TTE after MI, MR severity improved from baseline in approximately one-third, was stable in around half, with the remainder having worsening MR. Patients with persistent or worsening MR had worse clinical outcomes than those with improving MR.

## 1. Introduction

Mitral regurgitation (MR) is found in up to 29% of patients following acute myocardial infarction (MI) [1,2,3]. Approximately three-quarters of these patients have only mild MR [1,2]; however, even mild MR can be associated with poorer outcomes [4]. Long-term prognosis worsens in proportion to the severity of MR, such that patients with moderate or severe MR have only a 70% 5-year survival rate, even in the absence of significant LV impairment [1]. Due to a paucity of longitudinal studies, it is currently unclear whether the severity of MR observed immediately after acute MI changes with time, or whether such a change is associated with adverse prognosis. One of the largest recent studies in the modern era of drug-eluting stent treatment included only ST-elevation MI (STEMI) patients, and follow-up echocardiography was limited to 6–8 months [2]. Of particular interest is whether chronic ischaemic MR worsens, and whether there are baseline echocardiographic indicators of long-term MR severity. International guidelines do not currently recommend follow-up for patients on the basis of mild MR alone [5] and, thus, some patients with mild MR at baseline may develop undetected subclinical MR progression if they are not subject to follow-up.

We aimed to investigate the long-term course of MR and its effect on heart failure hospitalisation and mortality, in a retrospective longitudinal study involving follow-up echocardiography in patients with MR after an acute MI.

## 2. Materials and Methods

### 2.1. Patient Selection

The Queen Elizabeth Hospital Birmingham (QEHB) is a tertiary cardiac centre in the United Kingdom. Between 1 January 2016 and 31 December 2017, all consecutive patients admitted to QEHB with type 1 acute MI and treated by percutaneous coronary intervention (PCI) were retrospectively identified. For these patients, data were collected for every transthoracic echocardiography (TTE) performed after the index MI. The TTE performed pre-discharge for the index MI admission was classified as the “baseline” TTE. All subsequent TTEs were classified as “follow-up” TTEs; any follow-up TTEs performed ≤7 days after the index MI were excluded. Patients were considered for inclusion in the study if there was evidence of MR on the baseline TTE. Patients were then excluded if: (i) they had no further follow-up TTEs; (ii) the baseline TTE was >7 days after the index MI or (iii) they only had a single follow-up TTE, and this was ≤7 days after the index MI.

### 2.2. Data Collection

For all included patients, demographic data were collected from the time of the index MI admission. High-sensitivity troponin T levels were measured on a Roche Cobas E170^®^ (upper limit of detectable range 10,000 ng/L) at admission, and during the hospital stay, with the highest recorded troponin level for a patient classified as the “peak” troponin. Diagnoses of STEMI and non-ST elevation myocardial infarction (NSTEMI) were made by the admitting consultant cardiologist, according to standard international guideline definitions [6,7]. For analysis of long-term outcomes, evidence of heart failure was determined from admission history on hospital records, and mortality data were provided by the hospital records which are linked to the Office for National Statistics database, which tracks the living status of all patients in England and Wales via their NHS number. For the time-to-event analyses, heart failure or death was treated as a composite outcome; patients where there was no evidence of either event were censored at the date of data extraction (30 June 2021).

### 2.3. MR and Chamber Assessment

Transthoracic echocardiography was performed using CX-50 machines (Philips medical systems, Amsterdam, Netherlands). All studies included the minimum dataset [8] where possible, acquired by experienced echocardiographers accredited by the British Society of Echocardiography (BSE). Quantification of MR was performed using multiparametric assessment, as recommended in current guidelines of the American Society of Echocardiography and was categorized as none/trivial, mild, moderate, or severe, according to established criteria [9]. Dimensions were indexed according to the Mosteller calculation of body surface area (BSA). LV ejection fraction (LVEF) was derived from the indexed left ventricular end-diastolic (LVEDVi) and end-systolic (LVESVi) volumes, calculated by the Simpson’s biplane method. Indexed left atrial volume (LAVi) was calculated by averaging the volumes in the end-systolic frame of the apical 2- and 4-chamber views and indexing to BSA.

### 2.4. Intervention and Medical Treatment

All patients in the study underwent PCI, and were treated according to European guidelines on the management of AMI [6,7]. This included baseline treatment with loading and maintenance doses of aspirin and a P2Y12 receptor blocker, beta blockers, statins and angiotensin converting enzyme (ACE) inhibitors. Medication records from the time of each follow-up TTE were examined, to identify any evidence that heart failure medications had been optimised in the period since the previous TTE. “Medication optimisation” was defined as any uptitration of heart failure medication. In the case of mixed optimisation (simultaneous increase and decrease of several medications), a consensus was reached between two Cardiologists.

### 2.5. Statistical Analysis

Initially, the MR severity was compared between the baseline TTE, and the first and final follow-up TTEs for each patient, using Wilcoxon’s signed ranks test. Changes in MR severity were then further assessed using a repeated-measures approach. Each follow-up TTE was compared to the baseline TTE for the associated patient, and classified as either an increase, reduction or no change in MR severity, based on the direction of any change in the ordinal scale. Two dichotomous variables were then produced, one specifying whether each TTE represented an increase in MR severity (vs. no change or a reduction) compared to baseline, and one identifying reductions in MR severity (vs. no change or an increase). Generalized estimating equation (GEE) models were then produced for each of these outcomes, with the timing of the TTE, relative to baseline, as a continuous covariate. The TTE number was treated as a within-subject effect, with an AR (1) correlation structure assumed, in order to account for within-patient correlations. From these models, the intercept was interpreted as the magnitude of any immediate step-change in MR severity, whilst the gradient represented the rate of change in MR severity over subsequent follow-up. A similar analysis was also performed for the outcome of moderate-to-severe MR.

Since these analyses found no significant trends over time in MR severity after the initial step-change, subsequent analysis was based on comparisons of MR severity on the baseline vs. final follow-up TTE for each patient. As such, patients were divided into subgroups based on whether they had an increase, reduction, or no change in MR severity between these TTEs. Comparisons between these groups were performed using Jonckheere–Terpstra tests for continuous factors, so as to account for the ordering of the groups. For dichotomous variables, Mann–Whitney U tests were used, which treated the change in MR severity group as the dependent variable. Changes over time in other TTE parameters were quantified by calculating the difference between the baseline and final TTE for a patient, and dividing this by the time elapsed between these, to estimate the rate of change per year. Significant associations with MR severity were further visualised using two binary logistic regression models, with the dependent variables indicating whether the patient had either increases or decreases in MR severity (as previously described), and the variable of interest as a covariate.

Associations between MR progression and the composite outcome of death or heart failure (HF) admission were then assessed, using Kaplan–Meier curves and univariable Cox regression models. Initially, comparisons were made between groups based on the change in MR severity from the baseline vs. final follow-up TTE. However, a sensitivity analysis was also performed, which grouped patients by the change from baseline to the first follow-up TTE, within the subgroup of patients where this interval was less than 1 year, in order to assess the prognostic ability of short-term changes in MR severity.

Since only those patents with at least one follow-up TTE were included in the study, this cohort was then compared to those that were excluded, to test for evidence of selection bias. Continuous variables were assessed using Mann–Whitney U tests, with Fisher’s exact test used for categorical variables.

Continuous variables are reported as mean ± standard deviation (SD) where approximately normally distributed, with medians and interquartile ranges (IQRs) used otherwise. All analyses were performed using IBM SPSS 24 (IBM Corp. Armonk, NY, USA), with *p* < 0.05 deemed to be indicative of statistical significance throughout.

## 3. Results

### 3.1. Patient Demographics

**Figure 1:**
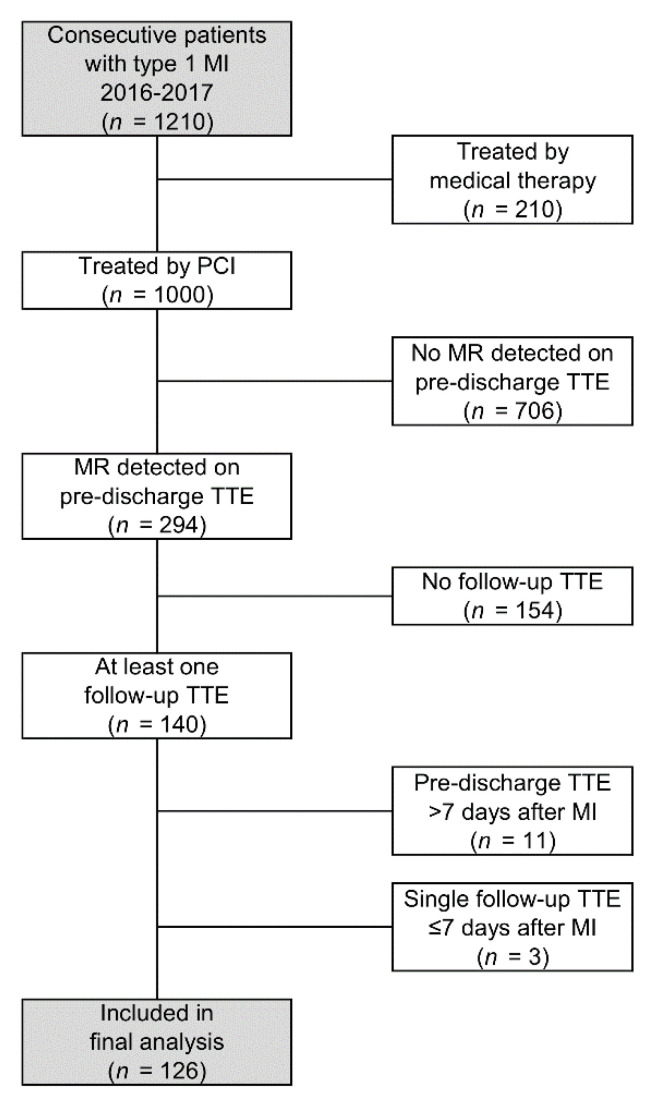
Study flowchart. MI = myocardial infarction; MR = mitral regurgitation; PCI= percutaneous coronary intervention; TTE= transthoracic echocardiography.

A total of *n* = 1000 consecutive patients underwent PCI for type 1 MI, of whom *n* = 294 were found to have MR on baseline TTE. Of these, *n* = 126 met the inclusion criteria, and were included in the analysis (Figure 1). These patients had a mean age at MI of 70.9 ± 11.4 years, 64% were male, and the majority had hypertension (69%). Further details of the cohort are reported in Table 1. In addition to the baseline TTE, data were available for a total of *n* = 230 follow-up TTEs (mean: 1.8 per patient). Most patients had a single follow-up TTE (56%), up to a maximum of seven follow-up TTEs. The final TTE was performed a median of 25 months (IQR: 7–45) after the baseline TTE, although there was considerable variability in this timing, with a range from 6 days to 63 months.

### 3.2. Changes in MR Severity

At the baseline TTE, most patients had mild MR (75%), with moderate and severe MR in 24% and 2%, respectively (Table 2).

At the first follow-up TTE, a median of 9 months (IQR: 3–23) after the baseline TTE, a significant improvement in MR was observed (*p* < 0.001), with 36% of patients having a reduction in MR severity, compared to 10% having an increase (Table 3a). In the subgroup of patients with additional follow up TTEs (*n* = 55), no further significant change in MR severity was observed between the first follow-up, and the final follow-up TTEs (*p* = 0.858, Table 3b), with 22% and 24% of patients having reductions and increases in MR severity, respectively. All echocardiography during follow-up was reviewed, and no change in mechanism for regurgitation was seen, for example, no cases were subject to de novo prolapse.

**Figure 2:**
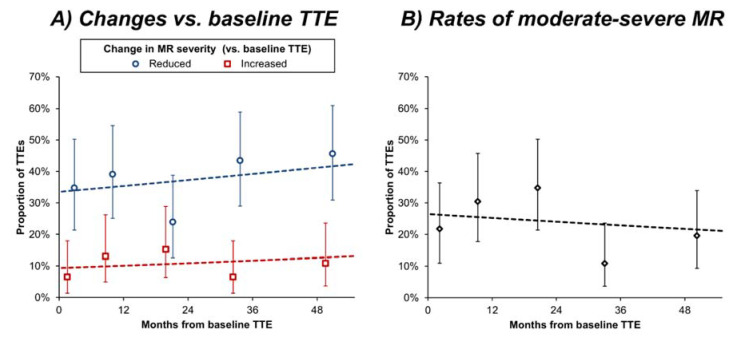
Changes in MR severity over time. Analyses are based on the *n* = 230 follow-up TTEs. Trends over time in MR were assessed using three generalized estimating equation models, each of which had a single covariate, indicating the timing of the TTE, relative to the baseline TTE. (**A**) visualises two of these models, with dependent variables specifying whether each TTE indicated an increase in MR severity, or a reduction in MR severity, respectively, compared to the baseline TTE. For (**B**) the dependent variable specified whether each TTE indicated moderate-to-severe MR. The resulting models are represented by broken lines. Points represent the observed rates of each outcome within subgroups of *n* = 46 consecutive TTEs (i.e., quintiles), and are plotted at the mean of the interval; whiskers represent 95% confidence intervals. MR = mitral regurgitation; TTE = transthoracic echocardiography.

Changes in MR severity, relative to the baseline TTE, were then assessed using a repeated-measures approach, which allowed data from all TTEs to be included in the analysis (Figure 2A). This returned results consistent with the above analysis, with an estimated 34% (95% confidence interval [CI]: 25–44%) of patients having immediate reductions, and 9% (95% CI: 5–17%) having immediate increases in MR severity after the baseline TTE. Thereafter, the proportions of patients with reductions (*p* = 0.383) or increases (*p* = 0.511) in MR severity (vs. baseline TTE) remained stable for the remainder of the follow-up period. Analysis of the proportion of patients with moderate-to-severe MR also returned similar results (Figure 2B), with an estimated rate of 27% (95% CI: 18–38%) immediately after the baseline scan, which remained stable over the remainder of follow-up (odds ratio: 0.94 per year, 95% CI: 0.76–1.15, *p* = 0.543).

### 3.3. Associations with Changes in MR Severity

Patients were then divided based on the change in MR severity from the baseline to final follow-up TTE; namely a reduction, no change, or an increase of at least one category of the ordinal scale. A range of factors were then compared between these groups, to identify potential predictors of changes in MR.

**Figure 3:**
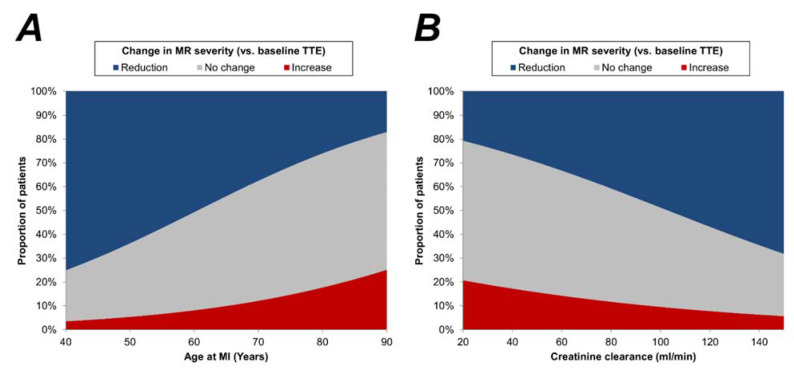
Associations between age/creatinine clearance and changes in MR severity. Plots are based on binary logistic regression models, with either the age (**A**) or creatinine clearance (**B**) as a continuous covariate. Two models were produced for each factor, with the dependent variable being reduction in MR severity (vs. increase or no change) in the first, and increase in MR severity (vs. reduction or no change) in the second. For all models, the changes in MR severity were assessed between the baseline and final TTE for each patient. The two models were then evaluated to estimate the rates of reductions and increases in MR severity for each value of the covariate, with the difference between these assumed to be the rate of “no change”. MI = myocardial infarction; MR = mitral regurgitation; TTE = transthoracic echocardiography.

This found patients with increases in MR severity to be significantly older at the time of MI, with a mean of 75.2 years, compared to 66.7 years in those with reductions MR severity (*p* = 0.003), and to have a significantly lower creatinine clearance (mean: 60 vs. 81 mL/min, *p* = 0.015, Table 4). These associations were further assessed using regression analysis, and the resulting models are visualised in Figure 3. For example, the models estimated that, for patients aged 50 years, MR severity would increase from the baseline TTE in 5% and reduce in 64% whilst, for those aged 80 years, these probabilities were 18% and 26%, respectively. In addition to the above, increasing MR severity was also found to be significantly more common in patients with hypertension (17% vs. 5%, *p* = 0.005). This analysis also assessed the associations between other TTE parameters and the change in MR severity. When measured at the baseline TTE, none of these parameters were found to be significant predictors of future changes in MR severity. However, increasing MR severity was found to be significantly associated with a corresponding increase in LVEDVi, with a median rate of change of +2.9 mL/m^2^ per year in those with an increase in MR severity, compared to −0.8 mL/m^2^ per year in those with a reduction in MR severity (*p* = 0.013). A similar trend was also observed for LVESVi (+3.5 vs. −1.2 mL/m^2^ per year, *p* = 0.016). There was no evidence of a significant association between the likelihood of MR progression and the coronary artery affected; however, the small number of patients with MR progression limited the statistical power of this analysis.

### 3.4. Associations with Patient Outcome

**Figure 4:**
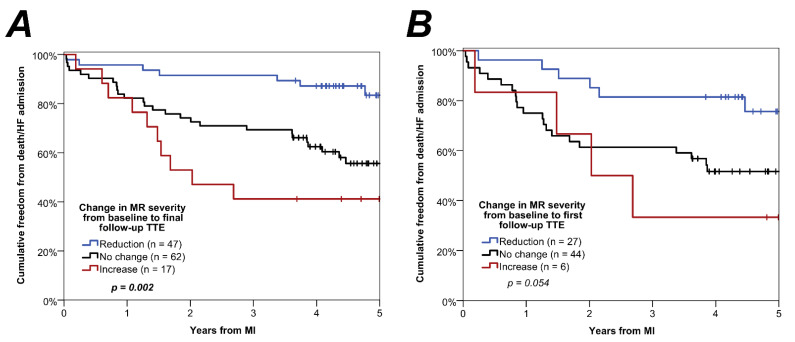
Kaplan–Meier curves of freedom from death/heart failure admission by change in MR severity. (**A**) groups all patients by the change in MR severity from the baseline TTE to the final follow-up TTE. (**B**) only includes those patients where the first follow-up TTE was within 1 year of the baseline TTE (*n* = 77), and groups these by the change in MR severity from baseline to the first follow-up TTE. *p*-Values are from univariable Cox regression models. HF = heart failure; MI = myocardial infarction; MR = mitral regurgitation; TTE = transthoracic echocardiography.

Patients were followed up for a median of 55 months (IQR: 46–61) after their MI, during which time *n* = 27 died, and *n* = 28 had an HF admission (*n* = 12 of whom subsequently died); hence, the composite outcome occurred in *n* = 43. Outcome rates were found to differ significantly by the change in MR severity from the baseline to final follow-up TTE (*p* = 0.002, Figure 4A). For those with a reduction in MR severity, the Kaplan–Meier estimated composite outcome rate was 16% at 5 years, compared to 44% (hazard ratio [HR]: 3.40, 95% CI: 1.47–7.83, *p* = 0.004) in those with no change, and 59% (HR: 5.64, 95% CI: 2.14–14.9, *p* < 0.001) in those with an increase in MR severity.

The analysis was then repeated considering the changes in MR severity between the baseline and first follow-up TTE, for the subgroup of patients where this interval was less than one year (*n* = 77), in order to assess whether early changes in MR severity could be predictive of long-term prognosis. This found similar trends to those previously described, with outcome rates at 5 years of 24%, 48%, and 67% for those with reductions, no change and increases in MR severity, respectively (Figure 4B). However, the difference between groups did not reach statistical significance (*p* = 0.054), which may be attributed to the smaller sample size in this analysis, particularly in the group with increasing MR severity (*n* = 6).

### 3.5. Assessment of Selection Bias

Since the aim of the study was to assess changes in MR severity, only those patients with at least one follow-up TTE were included. This resulted in more than half of the patients with MR following acute MI being excluded due to not having follow-up TTEs (57%; 168/294). To test for potential selection bias, the characteristics of these excluded patients were compared to those included in the main analysis (*n* = 126, Supplementary Table S1). This found the baseline demographics of the two groups to be generally similar, with the only significant difference being a lower rate of diabetes mellitus in the excluded patients (27% vs. 40%, *p* = 0.023). At the baseline TTE, the excluded patients had a higher mean LVEF (53 ± 15 vs. 50 ± 15%, *p* = 0.014), although no significant difference in MR severity was noted (moderate-to-severe: 23% vs. 25%, *p* = 0.769). However, 1-year mortality rates were found to be significantly higher in those who were excluded due to a lack of follow-up TTE (15%; 25/168), compared to those included in the final analysis (4%; 5/126; *p* = 0.003).

## 4. Discussion

This single-centre retrospective study is the first to investigate the course of MR and associated clinical outcomes over the longer term following acute MI, including both STEMI and NSTEMI patients. The study found that approximately one-third of patients see an improvement in MR severity by their first follow-up (median: 9 months), while approximately 1 in 10 patients with initially mild MR developed progression to moderate or severe MR. Furthermore, although it is well-established that MR following MI is associated with a worse outcome that is proportionate to the severity of regurgitation, our study suggests that this prognosis is modified by the subsequent change in MR. This raises the possibility that the trajectory of MR severity in the year following MI could be used to target patients for more intensive therapy, including targeted specialist care to ensure optimisation of medical therapy, together with careful case selection, either for surgical or transcatheter mitral intervention.

The present study adds to the previous literature investigating the trajectory of MR after acute MI, and finds that improvement generally occurs soon after MI. The main previous study included 390 patients undergoing echocardiography after STEMI and at 6 months from discharge [2]. Nishino et al. found that overall MR severity trends towards an improvement, with the proportion of patients with moderate or severe MR reducing from 10% to 5% and the proportion of patients with no MR increasing from 62% to 72% at 6 months. This early improvement in MR in both studies is likely due to post-infarction LV reverse remodelling, which occurs in up to one-third of patients following acute MI [10], a process accelerated by the impact of current drug therapy that is considered maximal in the first 12 months [11,12].

The patient group of most interest clinically are those with mild MR at baseline who progress to moderate or severe MR with time. Patients with mild MR are not routinely followed up on account of the MR alone [13], yet the 5-year follow-up data from this study reveals that patients with progression of mild MR had a significantly increased risk of HF hospitalisation and death, compared to patients with no change or improvement in MR. This is in keeping with previous studies, which have shown that clinical outcome is directly related to the severity of residual MR [1,2]. The translational aim of this work was to seek demographic, clinical, or echocardiographic markers to predict those patients most at risk of adverse long-term clinical outcomes in the early post-infarction period. While our study found the major drivers to progression of MR were older age, hypertension and lower creatinine clearance, these factors may be of limited clinical use as risk prediction tools, given the high prevalence of these conditions in the elderly population. A subgroup analysis of patients with exclusively mild MR at baseline (Supplementary Table S2) found no discernible difference in any clinical or echocardiographic risk factors amongst patients with and without progression of MR.

## 5. Conclusions

In this study of 126 consecutive patients having repeat TTE with MR following percutaneous intervention for MI at a large tertiary centre, most patients had mild MR, and a quarter had moderate or severe MR. At the first follow-up TTE, MR improved in many, but progressed in 1 in 10 patients. Change in MR severity affected prognosis: 16% with reduced MR reached the composite endpoint of death or heart failure hospitalisation at 5 years, compared to 44% with no change, and 59% with worsening MR. Other than older age, lower creatinine clearance and the presence of hypertension, no other imaging predictors were found to identify these individuals with the worst outcomes. The findings of this study suggest that a future prospective study may be warranted, in order to validate the conclusions.

## 6. Limitations

The primary limitation of the study is the potential for selection bias. This was a retrospective study, including a convenience sample of the TTEs performed at our centre. However, less than half of patients presenting with MR underwent a follow-up TTE. Therefore, we considered the possibility that those patients who had follow-up scans may have been more symptomatic, or considered higher risk by clinicians. In fact, when testing for such selection bias by comparing outcomes between the included and excluded patients, we found that 1-year mortality rates were higher in those patients with MR that were excluded, despite similar demographics in the two groups. Consequently, if some of these excess deaths in the excluded patients were as a result of undiagnosed early progression of MR, then rates of increasing MR severity may have been underestimated in our study.

In addition to the potential bias resulting from excluding patients, selection bias may also have influenced the timing and frequency of follow-up TTEs in those patients included in the study. Whilst most patients had only a single follow-up TTE, a maximum of seven TTEs was observed, and the timing of TTEs was highly variable. When assessing TTEs longitudinally, a repeated-measures methodology was used to prevent those patients with large numbers of TTEs having undue weight in the analysis. However, the inconsistent frequency and timing of follow-up TTEs may have meant that MR progression was more likely to be identified in those patients targeted by clinicians for regular follow-up, with diagnoses more likely to be missed or delayed in patients not selected for regular review. This again means that rates of increasing MR severity may have been underestimated in our study.

## Figures and Tables

**Table 1:** Patient demographics.

	*n*	Statistic
Age at MI (years)	126	70.9 ± 11.4
Sex (% male)	126	81 (64%)
BSA (m^2^)	126	1.84 ± 0.23
Hypertension	126	87 (69%)
Diabetes mellitus	126	50 (40%)
Creatinine clearance (mL/min)	126	70 ± 34
Peak troponin (ng/L)	126	647 (106–3430)
Type of MI		
NSTEMI	126	76 (60%)
STEMI	126	50 (40%)
Coronary artery involvement		
Left main coronary artery	126	9 (7%)
Left anterior descending coronary artery	126	97 (77%)
Left circumflex coronary artery	126	63 (50%)
Right coronary artery	126	61 (48%)
Baseline TTE		
Days from MI to baseline TTE	126	2 (1–3)
LVEDVi (mL/m^2^)	115	50 (41–69)
LVESVi (mL/m^2^)	115	27 (17–39)
LAVi (mL/m^2^)	122	32 (25–44)
LVEF (%)	125	50 ± 15
Follow-up TTEs		
Number of follow-up TTEs	126	
1		71 (56%)
2		24 (19%)
3		20 (16%)
4		6 (5%)
5		4 (3%)
6		0 (0%)
7		1 (1%)
Months from baseline to final TTE	126	25 (7–45)
Evidence of medication optimisation *	120	60 (50%)

Data are reported as *n* (%), mean ± SD, or as median (IQR), as applicable. * Evidence of medication optimisation in the notes taken at any follow-up visit. BSA = body surface area; LVED(S)Vi = left ventricular end-diastolic (systolic) volume index; LVEF = left ventricular ejection fraction; LAVi = left atrial volume index; MI = myocardial infarction; MR = mitral regurgitation; TTE= transthoracic echocardiography; (N)STEMI = (Non-)ST-elevation MI.

**Table 2:** Changes in MR severity.

	Baseline TTE	First Follow-up TTE	Final Follow-up TTE
Months from baseline TTE	-	9 (3–23)	25 (7–45)
MR severity			
None	-	34 (27%)	34 (27%)
Mild	94 (75%)	60 (48%)	60 (48%)
Moderate	30 (24%)	29 (23%)	28 (22%)
Severe	2 (2%)	3 (2%)	4 (3%)
Change in MR severity (vs. baseline) *			
Reduced	-	45 (36%)	47 (37%)
No change	-	68 (54%)	62 (49%)
Increased	-	13 (10%)	17 (13%)
*p*-Value		***p* < 0.001**	***p* < 0.001**

Data are reported as *n* (%), or as median (interquartile range), as applicable. In those patients with only a single follow-up TTE, the MR severity at the first and final follow-up TTE are derived from the same TTE. *p*-Values are comparisons of the MR severity on the follow-up vs. baseline TTEs using Wilcoxon’s signed ranks test, and bold p-values are significant at *p* < 0.05. * Changes of at least one category in the ordinal scale, relative to the baseline echo. MR = mitral regurgitation; TTE = transthoracic echocardiography.

**Table 3:** (a) MR severity on baseline TTE v final follow-up TTE. (b) MR severity on first follow-up TTE vs. final follow-up TTE.

**(a)**				
**MR Severity:** **Baseline TTE**	**MR Severity: Final Follow-up TTE**			
	**None**	**Mild**	**Moderate**	**Severe**
*None*	-	-	-	-
*Mild*	30	47	15	2
*Moderate*	4	13	13	0
*Severe*	0	0	0	2
**(b)**				
**MR Severity:** **First Follow-up TTE**	**MR Severity: Final Follow-up TTE**			
	**None**	**Mild**	**Moderate**	**Severe**
*None*	8	6	0	0
*Mild*	5	14	6	1
*Moderate*	1	6	8	0
*Severe*	0	0	0	0

Green cells represent a reduction in MR severity, with red cells representing an increase. (a) An overall comparison found a significant reduction in MR severity from baseline to the final follow-up TTE (Wilcoxon’s signed ranks test: *p* < 0.001). (b) Only those patients with more than one follow-up TTE are included (*n* = 55). An overall comparison found no significant change in MR severity from the first to final follow-up TTE (Wilcoxon’s signed ranks test: *p* = 0.858). MR = mitral regurgitation; TTE = transthoracic echocardiography.

**Table 4:** Associations between baseline factors and the change in MR severity between baseline and the final TTE.

		Change in MR Severity from Baseline to Final TTE			
	*n*	Reduction	No change	Increase	*p*-Value
Age at MI (years)	126	66.7 ± 11.5	72.9 ± 11.5	75.2 ± 6.6	**0.003**
Sex	126				0.660
Female		16 (36%)	26 (58%)	3 (7%)	
Male		31 (38%)	36 (44%)	14 (17%)	
BSA (m^2^)	126	1.89 ± 0.24	1.81 ± 0.22	1.81 ± 0.25	0.065
Hypertension	126				**0.005**
No		21 (54%)	16 (41%)	2 (5%)	
Yes		26 (30%)	46 (53%)	15 (17%)	
Diabetes mellitus	126				0.173
No		32 (42%)	35 (46%)	9 (12%)	
Yes		15 (30%)	27 (54%)	8 (16%)	
Creatinine clearance (mL/min)	126	81 ± 40	64 ± 29	60 ± 22	**0.015**
Peak troponin (ng/L)	126	892 (86–3979)	546 (191–2837)	730 (38–3926)	0.736
Type of MI	126				0.195
NSTEMI		26 (34%)	37 (49%)	13 (17%)	
STEMI		21 (42%)	25 (50%)	4 (8%)	
Evidence of medication optimisation *	120				0.577
No		23 (38%)	29 (48%)	8 (13%)	
Yes		20 (33%)	31 (52%)	9 (15%)	
Baseline TTE parameters					
LVEDVi (mL/m^2^)	126	51 (42–61)	50 (38–72)	55 (41–89)	0.630
LVESVi (mL/m^2^)	126	25 (17–35)	27 (16–42)	27 (22–61)	0.317
LAVi (mL/m^2^)	126	31 (24–41)	32 (24–44)	36 (30–48)	0.344
LVEF (%)	125	52 ± 13	50 ± 15	45 ± 19	0.196
Change in TTE parameters from baseline to final follow-up TTE **					
Change in LVEDVi (mL/m^2^ per year) **	110	−0.8 (−3.4, 2.5)	2.5 (−3.9, 13.1)	2.9 (2.1, 15.4)	**0.013**
Change in LVESVi (mL/m^2^ per year) **	110	−1.2 (−3.1, 0.7)	0.2 (−6.3, 6.2)	3.5 (0.8, 14.6)	**0.016**
Change in LVEF (PP per year) **	118	0.7 (−2.4, 4.8)	0.0 (−4.1, 7.4)	−0.3 (−5.9, 3.6)	0.316

Continuous variables are reported as mean ± standard deviation, or as median (interquartile range), with p-values from Jonckheere–Terpstra tests. Dichotomous variables are reported as n (row %), with p-values from Mann–Whitney U tests, treating the change in MR severity as the dependent variable. Bold p-values are significant at *p* < 0.05. * Evidence of medication optimisation in the notes taken at any follow-up visit. ** For each patient, the rate of change in the parameter was estimated by dividing the change from the baseline to final TTE by the time between them; the resulting values were then compared between groups. BSA = body surface area; LVED(S)Vi = left ventricular end-diastolic (systolic) volume index; LVEF = left ventricular ejection fraction; LAVi = left atrial volume index; MI = myocardial infarction; MR = mitral regurgitation; PCI = percutaneous coronary intervention; PP = percentage point; TTE = transthoracic echocardiography; (N)STEMI = (Non-)ST-elevation MI.

## Data Availability

The data presented in this study are available on request from the corresponding author.

## Supplementary Materials

The following supporting information can be downloaded at: https://www.mdpi.com/article/10.3390/jcm11040965/s1, Table S1: Comparisons between patients included (*n* = 126) vs. excluded (*n* = 168) from the study derived from all patients who had MR following MI (*n* = 294); Table S2: Associations between baseline factors and the change in MR severity between baseline and the final echo for patients with mild MR at baseline.
