# Supplementary material for: A Longitudinal Study of Mitral Regurgitation Detected after Acute Myocardial Infarction
Source: J Clin Med. 2022 Feb 13;11(4):965. doi: 10.3390/jcm11040965 (PMC8880345; doi:10.3390/jcm11040965)
Supplement: Supplementary file 1 [file jcm-11-00965-s001.zip › Supplementary Table S1.pdf]

Supplementary Table S1 – Comparisons between patients included (n = 126) vs. excluded (n = 168) from the study derived from all patients who had MR following MI (n = 294)

	Included (n=126)		***Excluded (n=168)***		
	n	*Statistic*	*n*	*Statistic*	**p-Value**
Age at MI (years)	126	70.9 ± 11.4	168	69.7 ± 13.1	0.572
Sex (% male)	126	81 (64.3%)	168	108 (64.3%)	1.000
Hypertension	126	87 (69.0%)	167	100 (59.9%)	0.112
Diabetes mellitus	126	50 (39.7%)	168	45 (26.8%)	**0.023**
Creatinine clearance (ml/min)	126	70 ± 34	166	69 ± 32	0.799
Peak troponin (ng/L)	126	647 (106-3430)	166	876 (218-3158)	0.285
NSTEMI	126	76 (60.3%)	168	92 (54.8%)	0.405
One-year mortality	126	5 (4.0%)	168	25 (14.9%)	**0.003**
**Baseline TTE**					
LVEDVi (ml/m^2)	115	50 (41-69)	149	48 (40-57)	0.130
LVESVi (ml/m^2)	115	27 (17-39)	149	21 (16-29)	**0.009**
LAVi (ml/m^2)	122	32 (25-44)	67	37 (29-53)	**0.025**
LVEF (%)	125	50 ± 15	166	53 ± 15	**0.014**
MR severity	126		168		0.769*
Mild		94 (74.6%)		129 (76.8%)	
Moderate		30 (23.8%)		32 (19.0%)	
Severe		2 (1.6%)		7 (4.2%)	

*Continuous variables are reported as mean ± standard deviation, or as median (interquartile range), with p-values from Mann-Whitney U tests. Dichotomous variables are reported as n (column %), with p-values from Fisher's exact tests. Bold p-values are significant at p<0.05. *p-Value from Mann-Whitney U test, as the factor is ordinal. LVED(S)Vi=left ventricular end-diastolic (systolic) volume index; LVEF=left ventricular ejection fraction; LAVi=left atrial volume index; MI=myocardial infarction; MR=mitral regurgitation; TTE= transthoracic echocardiography; NSTEMI=Non-ST-elevation MI.*
