# Supplementary material for: A Longitudinal Study of Mitral Regurgitation Detected after Acute Myocardial Infarction
Source: J Clin Med. 2022 Feb 13;11(4):965. doi: 10.3390/jcm11040965 (PMC8880345; doi:10.3390/jcm11040965)
Supplement: Supplementary file 1 [file jcm-11-00965-s001.zip › Supplementary Table S2.pdf]

Supplemental Table S2 - Associations between baseline factors and the change in MR severity between baseline and the final echo for patients with mild MR at baseline

	n	Change in MR severity from baseline to final TTE<br>*Reduction/ no change*	*Increase*	*p-Value*
Age at MI (Years)	94	69.4 ± 11.7	75.2 ± 6.6	0.076
Sex	94			0.253
Female		26 (90%)	3 (10%)	
Male		51 (78%)	14 (22%)	
BSA (m^2)	94	1.87 ± 0.23	1.81 ± 0.25	0.223
Hypertension	94			0.223
No		23 (92%)	2 (8%)	
Yes		54 (78%)	15 (22%)	
Diabetes mellitus	94			0.788
No		45 (83%)	9 (17%)	
Yes		32 (80%)	8 (20%)	
Creatinine clearance (ml/min)	94	77 ± 37	60 ± 22	0.135
Peak troponin (ng/L)	94	592 (120-2878)	730 (38-3926)	0.662
Type of MI	94			0.270
NSTEMI		45 (78%)	13 (22%)	
STEMI		32 (89%)	4 (11%)	
Baseline TTE Parameters				
LVEDVi (ml/m^2)	86	48 (38-59)	55 (41-89)	0.210
LVESVi (ml/m^2)	86	23 (15-35)	27 (22-61)	0.149
LAVi (ml/m^2)	92	30 (23-39)	36 (30-48)	0.083
LVEF (%)	94	52 ± 15	45 ± 19	0.127

Only those patients with mild MR at baseline were included in the analysis (n=94). Continuous variables are reported as mean ± standard deviation, or as median (interquartile range), with p-values from Mann-Whitney U tests. Dichotomous variables are reported as n (row %), with p-values from Fisher's exact tests. Bold p-values are significant at p<0.05. BSA=body surface area; LVED(S)Vi=left ventricular end-diastolic (systolic) volume index; LVEF=left ventricular ejection fraction; LAVi=left atrial volume index; MI=myocardial infarction; MR=mitral regurgitation; TTE= transthoracic echocardiography; (N)STEMI=(Non-)ST-elevation MI.
